# Efficacy of ARB/HCTZ Combination Therapy in Uncontrolled Hypertensive Patients Compared with ARB Monotherapy: A Meta-Analysis

**DOI:** 10.1155/2021/6670183

**Published:** 2021-04-27

**Authors:** Linlin Ma, Keyang Zheng, Jiafu Yan, Wenli Cheng

**Affiliations:** ^1^Department of Hypertension, Beijing Anzhen Hospital, Capital Medical University, Beijing, China; ^2^Capital Medical University, Beijing, China

## Abstract

**Objective:**

To evaluate the efficacy of combination of angiotensin receptor blocker (ARB) with hydrochlorothiazide (HCTZ) compared to ARB alone in patients with uncontrolled hypertension via a systematic review and meta-analysis.

**Methods:**

We searched databases till July 2019 using relevant search terms. We included articles that were randomised controlled trials (RCTs) comparing ARB/HCTZ with ARB for a duration of at least 4 weeks and reported on the efficacy or safety. Meta-analyses for efficacy outcomes were performed. In addition, groups given different concentrations of HCTZ (12.5 and 25 mg) were analysed separately.

**Results:**

Sixteen RCTs (12,055 participants) were included. Overall, ARB/HCTZ combination therapy (both 12.5 and 25 mg HCTZ combination) resulted in better sitting systolic and diastolic blood pressure control than ARB alone (mean difference (95% confidence interval (CI): −5.69 [−6.66, −4.73] for 12.5 mg and −9.10 [−11.78, −6.42] for 25 mg and mean difference (95% CI): −2.91 [−3.31, −2.51] for 12.5 mg and −4.16 [−4.75, −3.58] for 25 mg). ARB/HCTZ combination therapy resulted in a higher rate of target blood pressure achievement compared to ARB alone (risk ratio (95% CI): 1.50 [1.42, 1.59]). ARB/HCTZ combination therapy had similar rates of total adverse events (AEs) and severe AEs compared to ARB alone.

**Conclusion:**

ARB/HCTZ combination therapy is more efficacious for controlling blood pressure, and combination with a low concentration of HCTZ has similar AEs compared to ARB alone. Clinicians should consider adding HCTZ in the medication regime of patients with uncontrolled hypertension using ARB, if their clinical profile allows.

## 1. Introduction

The prevalence of hypertension is increasing worldwide [1]. Adequate control of blood pressure (BP) is therefore of paramount importance [2]. A high BP is associated with a higher risk of developing myocardial infarction, heart failure, stroke, and kidney disease [2].

Initial combination therapy for patients with hypertension has included a diuretic in combination with a *β* blocker, angiotensin-converting enzyme inhibitor (ACEI), or angiotensin receptor blocker (ARB) [3]. Studies have established that the addition of a thiazide‐type diuretic, such as hydrochlorothiazide (HCTZ), to ARB therapy enhances the BP-lowering ability and increases the proportion of patients who achieve goal BP with initial treatment [3]. Although several primary studies have reported on combination therapy with ARB/HCTZ in adult patients with primary hypertension, the overall efficacy and safety of combination ARB/HCTZ therapy in patients with uncontrolled hypertension on renin angiotensin system inhibitor (RASI) therapy has not been evaluated.

Therefore, we aim to summarize the efficacy of additional ARB/HCTZ in patients with uncontrolled hypertension on RASI therapy via a systematic review and meta-analysis. We hope the results will help clinicians choose the appropriate therapy to manage patients' hypertension while reducing the risk of adverse events (AEs).

## 2. Materials and Methods

### 2.1. Data Sources and Search Strategies

Studies were identified by a literature search of MEDLINE (PubMed), the Cochrane Central Register of Controlled Trials (CENTRAL), the Cochrane Database of Systematic Reviews (CDSR), Database of Abstracts of Reviews of Effects (DARE), and EMBASE till July 2019. The search strategies were as follows: (ARB OR “angiotensin II receptor blockers” OR valsartan OR losartan OR telmisartan OR irbesartan OR tasosartan OR candesartan OR eprosartan OR azilsartan OR olmesartan OR fimasartan) AND (“thiazide diuretics” OR hydrochlorothiazide OR indapamide) AND (hypertension OR hypertensive OR “blood pressure”) AND (random OR randomly OR randomized). The searched studies were limited to clinical trials in humans.

### 2.2. Eligibility and Exclusion Criteria for Study Selection

We included studies that were randomised controlled trials (RCTs) that included adult patients with uncontrolled hypertension receiving RASI therapy and compared the ARB/HCTZ combination therapy with comparators such as ARB or ARB + placebo, with or without background therapy, had a study duration more than or equal 4 weeks, and reported at least one clinical outcome of interest. We excluded studies that were not RCTs, were conference abstracts, or were published in a language other than English.

### 2.3. Data Extraction

Two independent investigators extracted the data. We extracted publication data (title, first author, and year of publication), study design, baseline characteristics of the study population (sample size, age, sex ratio, duration of hypertension, baseline systolic blood pressure (SBP), and diastolic blood pressure (DBP)), drug regimen, treatment duration, efficacy outcomes (changes in sitting SBP (SiSBP), sitting DBP (SiDBP), response rate, and BP control rate), and safety outcomes (incidence of AEs) from each study. Some studies applied two concentrations of HCTZ (12.5 mg and 25 mg), and the data from these two groups in these studies were all extracted.

### 2.4. Assessment of Study Quality and Risk of Publication Bias

The quality of all included studies was assessed by the Cochrane risk-of-bias tool including selection bias, performance bias, detection bias, attrition bias, reporting bias, and others [4]. For assessment of publication bias for the primary outcome, we used funnel plot analysis and Egger's test (if possible).

### 2.5. Statistical Analysis

For continuous values, including the changes in SiSBP and SiDBP from baseline, effect sizes were estimated using meta-analysis as a weighted mean difference (WMD) with 95% confidence interval (95% CI). For categorical outcomes, such as the number of participants who experienced AEs and those with a successful response, the pooled risk ratio (RR) with its associated 95% CI was calculated using meta-analysis. The *I*^2^ statistic was used to calculate the extent of heterogeneity across the selected studies. The meta-analysis was conducted using RevMan 5.3 (The Cochrane Collaboration, Oxford, UK).

## 3. Results

### 3.1. Search Results

**Figure 1:**
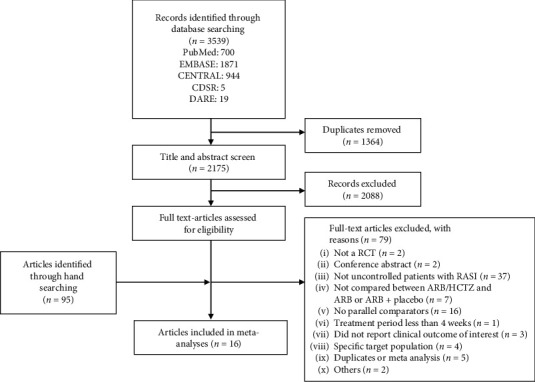
Flow chart of article selection through the review process. Abbreviations: randomised controlled trial (RCT), angiotensin II receptor blocker (ARB), hydrochlorothiazide (HCTZ), the Cochrane Central Register of Controlled Trials (CENTRAL), the Cochrane Database of Systematic Reviews (CDSR), Database of Abstracts of Reviews of Effects (DARE).

As shown in Figure 1, our searches identified 3539 studies, of which 2175 were potentially relevant based on title and abstract screening. Records (*n* = 2088) were excluded after reading the title and abstract, and 95 articles were identified by reading the full text. A total of 16 studies were found to be suitable for the meta-analysis. [5–20].

### 3.2. Study Characteristics

Of the 16 studies, 3 studies were intercontinental, 5 studies were in the European setting, 4 studies were in the United States and Canada, and 4 studies were in Asian countries. The duration of intervention ranged from 4 weeks to 12 weeks, and the majority of the patients were male in all studies. All 16 studies used a combination therapy with 12.5 mg HCTZ, and 6 studies also tested combination therapy with 25 mg HCTZ. Additional characteristics of the studies are presented in Supplementary Table 1.

### 3.3. Quality of Included Studies and Risk of Publication Bias

We performed a quality assessment for each study using the Cochrane risk-of-bias tool. The risk of bias analysis is summarized in Supplementary Figure 1. There were 12 studies not clearly describing the methods of random sequence generation and/or allocation concealment (selection bias). One study showed a high risk of performance and detection bias. Risk of performance bias was unclear in four trials, and the risk was low for the other studies. All other bias risks were low for all studies.

The funnel plot and Egger's test showed no evidence of publication bias for all efficacy outcomes (*p*=0.3352 for SiSBP, *p*=0.1204 for SiDBP, *p*=0.3364 for response rate, and *p*=0.0676 for BP goal achievement rate) and AEs (only 9 studies were included, and Egger's test could not be performed). The results of the funnel plot analysis are depicted in Supplementary Figure 2.

### 3.4. Efficacy Outcomes

#### 3.4.1. SiSBP and SiDBP

**Figure 2:**
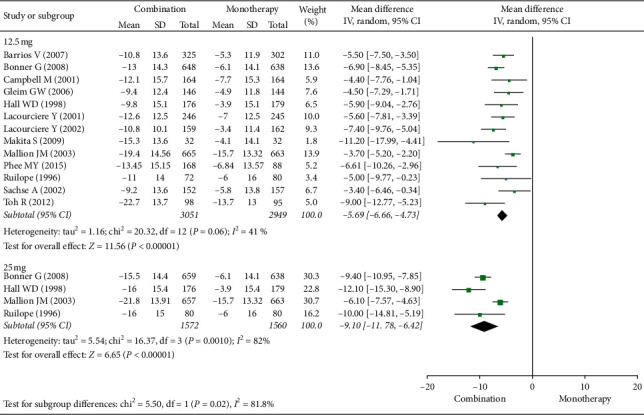
Forest plot for sitting systolic blood pressure (SiSBP) changes.

Figure 2 shows that, with the ARB/HCTZ combination, the reduction in SiSBP was greater compared with ARB alone. For combination therapy with 12.5 mg HCTZ, the mean difference (95% CI) was −5.69 (−6.66, −4.73) from 13 studies with an *I*^2^ of 41%. For combination therapy with 25 mg HCTZ, the mean difference (95% CI) was −9.10 (−11.78, −6.42) from 4 studies with an *I*^2^ of 82%. The reduction in SiSBP with combination therapy including 25 mg HCTZ was significantly greater than that with combination therapy with 12.5 mg HCTZ (*p*=0.02).

**Figure 3:**
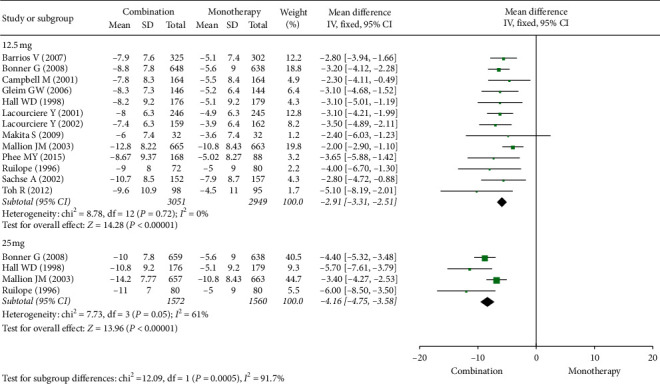
Forest plot for sitting diastolic blood pressure (SiDBP) changes.

Figure 3 shows that, with use of the ARB/HCTZ combination, the reduction in SiDBP was greater than with ARB alone. For combination therapy with 12.5 mg HCTZ, the mean difference (95% CI) was −2.91 (−3.31, −2.51) from 13 studies with an *I*^2^ of 0%. For combination therapy with 25 mg HCTZ, the mean difference (95% CI) was −4.16 (−4.75, −3.58) from 4 studies with an *I*^2^ of 61%. The reduction in SiSBP with combination therapy with 25 mg HCTZ was significantly greater than that with combination therapy with 12.5 mg HCTZ (*p*=0.0005).

#### 3.4.2. Response Rate and BP Goal Achievement Rate

**Figure 4:**
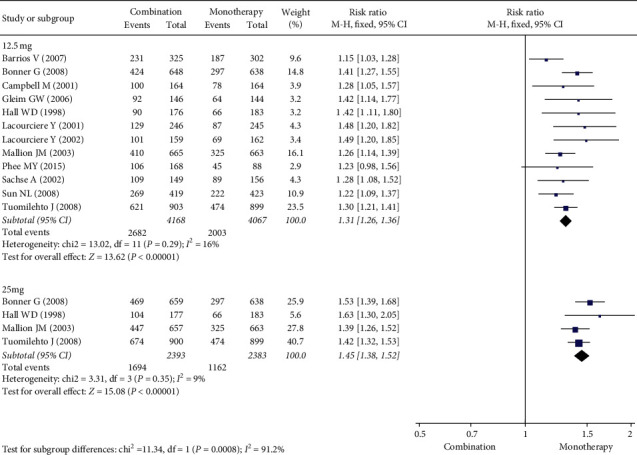
Forest plot for response rate.

Figure 4 shows that, with the use of the ARB/HCTZ combination, the response rate was higher than with ARB alone. With the combination therapy with 12.5 mg HCTZ, the RR (95% CI) was 1.50 (1.42, 1.59) from 12 studies with an *I*^2^ of 69%. With the combination therapy with 25 mg HCTZ, the RR (95% CI) was 1.78 (1.63, 1.95) from 3 studies with an *I*^2^ of 1%. The response rate with combination therapy with 25 mg HCTZ was significantly higher than that with combination therapy with 12.5 mg HCTZ (*p*=0.001).

**Figure 5:**
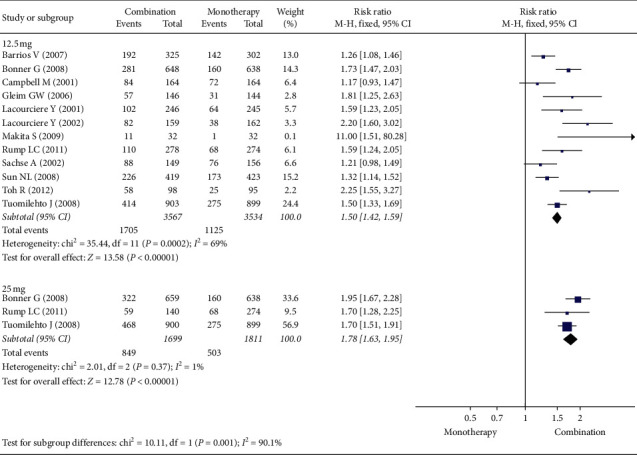
Forest plot for BP goal achievement rate.

Figure 5 shows that, with the ARB/HCTZ combination, the rate of BP goal achievement was similar compared with that achieved with ARB alone. With the combination therapy with 12.5 mg HCTZ, the RR (95% CI) was 1.08 (0.72, 1.64) from 7 studies with an *I*^2^ of 0%. With the combination therapy with 25 mg HCTZ, the RR (95% CI) was 1.16 (0.70, 1.90) from 4 studies with an *I*^2^ of 0%.

### 3.5. Safety Outcomes

**Figure 6:**
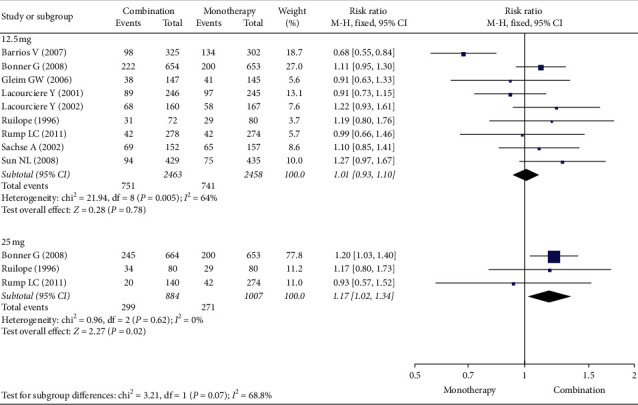
Forest plot for total adverse events (AEs).

Figure 6 shows that the total incidence of AEs with the combination therapy with 12.5 mg HCTZ was similar to that with ARB alone (RR (95% CI): 1.01 [0.93, 1.10], *I*^2^:64%, 9 studies), while the combination therapy with 25 mg HCTZ was associated with a significantly higher RR (RR (95% CI): 1.17 [1.02, 1.34], *I*^2^:0%, 3 studies). As shown in Supplementary Figure 3, in terms of drug-related AEs, with the ARB/HCTZ combination, patients experienced a higher incidence rate with both concentrations of HCTZ than with ARB alone (RR (95% CI): 1.39 [1.12, 1.72], *I*^2^: 0%, 9 studies for 12.5 mg HCTZ; RR (95% CI): 1.52 [1.01, 2.29], *I*^2^: 0%, 2 studies for 25 mg HCTZ). As shown in Supplementary Figure 4, with the ARB/HCTZ combination, the rate of serious AEs for both concentrations of HCTZ was comparable to that with ARB alone (RR (95% CI): 0.77 [0.47, 1.27], *I*^2^: 0%, 9 studies for 12.5 mg HCTZ; RR (95% CI): 0.70 [0.35, 1.40], *I*^2^: 0%, 3 studies for 25 mg HCTZ). Last, as shown in Supplementary Figure 5, with use of the ARB/HCTZ combination, the rate of discontinuation due to AEs was similar to that with the use of ARB alone (RR (95% CI): 1.08 [0.72, 1.64], *I*^2^: 0%, 7 studies for 12.5 mg HCTZ; RR (95% CI): 1.16 [0.70, 1.90], *I*^2^: 0%, 4 studies for 25 mg HCTZ).

## 4. Discussion

We conducted a systematic review and meta-analysis to evaluate the efficacy and safety of using the combination of ARB/HCTZ compared to ARB alone in patients with uncontrolled hypertension. This is the first study conducted to allow clinicians to understand the impact of additional HCTZ in patients with uncontrolled hypertension on RASI therapy. We found that the ARB/HCTZ combination was more efficacious and also safe compared to the use of ARB alone.

Combination of ARB/HCTZ resulted in a lower SiSBP and SiDBP compared to ARB alone. Moreover, when the combination therapy included 25 mg HCTZ, it resulted in a lower SiSBP and SiDBP compared with combination therapy including 12.5 mg HCTZ. Combination therapy achieved a higher response rate compared to ARB alone, while the BP goal achievement rates were comparable with the two therapies. In terms of the safety profile, addition of 12.5 mg HCTZ did not increase the rate of AEs, which is similar to the findings of other studies, such as the review described by Flack et al., whereby ARB/HCTZ combination had a higher potency but similar safety profile [21]. However, addition of 25 mg HCTZ resulted in an increase in AEs. Moreover, combination therapy showed an increase in drug-related AEs compared with ARB alone. However, the incidence of severe AEs and discontinuation due to AEs were comparable between two therapies. Therefore, from the results for both the efficacy and safety of ARB/HCTZ combination therapy, clinicians should take into account a patient's clinical profile, especially his/her comorbidities, before prescribing the combination therapy [22].

Our study has a few limitations. First, we only included English language articles. However, during our search, we did not encounter any articles that were not published in English. Second, our studies were limited to those with an intervention period of at least 4 weeks. This meant that studies with a maximum duration less than 4 weeks were excluded. Third, our study lacked individual patient data, which prevented more detailed exploration of heterogeneity.

## 5. Conclusions

Using a combination of ARB with a low concentration of HCTZ afforded better BP control without additional AEs compared with using ARB alone in patients with uncontrolled hypertension. Clinicians should consider addition of a diuretic to improve BP and control for future complications of hypertension.

## Data Availability

The data that support the findings of this study are available from the corresponding author upon reasonable request.

## Supplementary Materials

Supplementary Materials Supplementary Table 1: study characteristics of included studies. Supplementary Figure 1: summary of bias of the included studies by the Cochrane risk-of-bias tool. Supplementary Figure 2: funnel plot with Egger's test for assessing the risk of publication bias. Supplementary Figure 3: forest plot for drug-related adverse events (AEs). Supplementary Figure 4: forest plot for severe adverse events (SAEs). Supplementary Figure 5: forest plot for discontinuation due to adverse events (AEs).
