# Supplementary material for: Efficacy of ARB/HCTZ Combination Therapy in Uncontrolled Hypertensive Patients Compared with ARB Monotherapy: A Meta-Analysis
Source: Int J Hypertens. 2021 Apr 27;2021:6670183. doi: 10.1155/2021/6670183 (PMC8096582; doi:10.1155/2021/6670183)
Supplement: Supplementary Materials — Supplementary Table 1: study characteristics of included studies. Supplementary Figure 1: summary of bias of the included studies by the Cochrane risk-of-bias tool. Supplementary Figure 2: funnel plot with Egger's test for assessing the risk of publication bias. Supplementary Figure 3: forest plot for drug-related adverse events (AEs). Supplementary Figure 4: forest plot for severe adverse events (SAEs). Supplementary Figure 5: forest plot for discontinuation due to adverse events (AEs). [file 6670183.f1.zip › 6670183.f1/Supplementary Figure 2 (1).pdf]

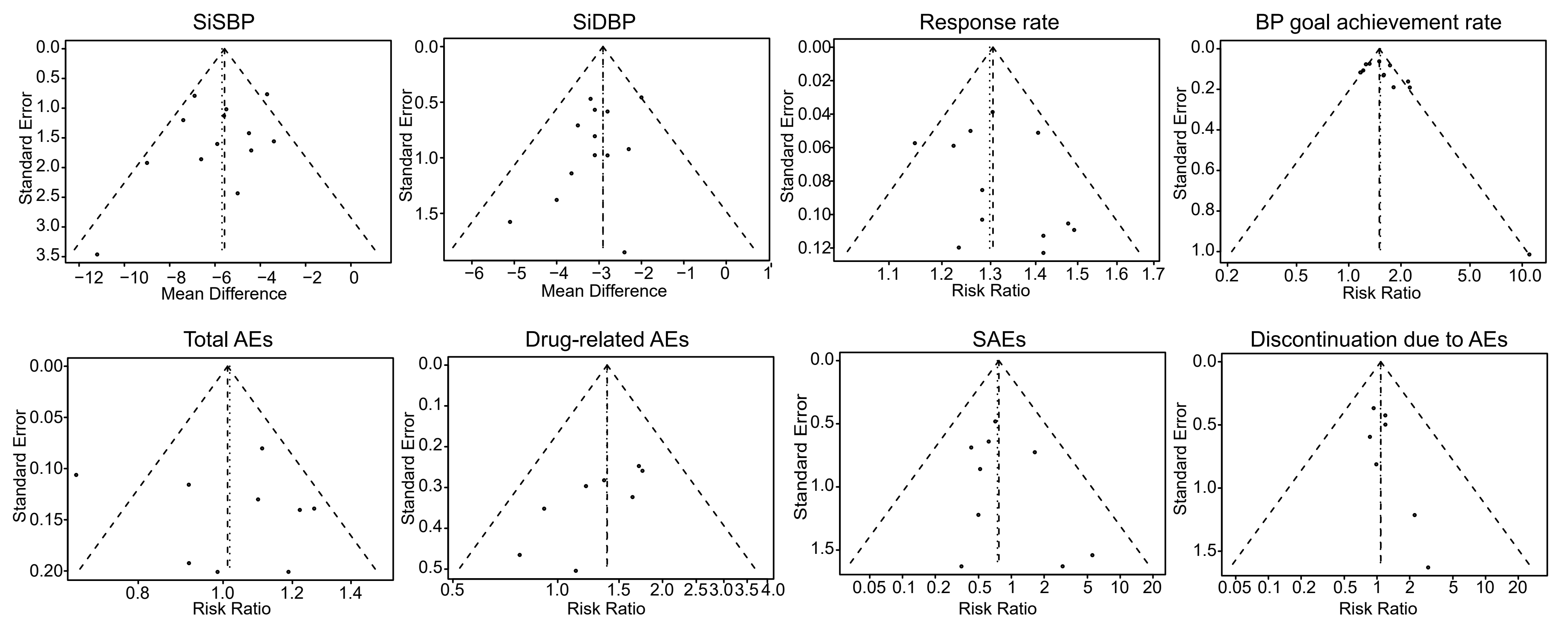
SiSBP
SiDBP
Response rate
BP goal achievement rate
Total AEs
Drug-related AEs
SAEs
Discontinuation due to AEs
Standard Error
Mean Difference
Risk Ratio
