# Supplementary material for: Efficacy of ARB/HCTZ Combination Therapy in Uncontrolled Hypertensive Patients Compared with ARB Monotherapy: A Meta-Analysis
Source: Int J Hypertens. 2021 Apr 27;2021:6670183. doi: 10.1155/2021/6670183 (PMC8096582; doi:10.1155/2021/6670183)
Supplement: Supplementary Materials — Supplementary Table 1: study characteristics of included studies. Supplementary Figure 1: summary of bias of the included studies by the Cochrane risk-of-bias tool. Supplementary Figure 2: funnel plot with Egger's test for assessing the risk of publication bias. Supplementary Figure 3: forest plot for drug-related adverse events (AEs). Supplementary Figure 4: forest plot for severe adverse events (SAEs). Supplementary Figure 5: forest plot for discontinuation due to adverse events (AEs). [file 6670183.f1.zip › 6670183.f1/Supplementary Table 1.pdf]

Supplementary Table 1. Study characteristics of included studies

Study	Groups	Design	Country	Does of RASI before study (uncontrolled patients)	Dose of ARB	Dose of HCTZ	Number of patients	Intervention duration	Age (mean±SD)	Male	BMI (mean±SD)	Weight (mean±SD)	Height (mean ± SD)	Baseline pulse rate (mean ± SD)	Caucasian	Smoking habit	History of diabetes	Duration of hypertension (mean ± SD)	Baselinesitting systolic BP (mean ± SD)	Baselinesitting diastolic BP (mean ± SD)
1st author (publication year)							(randomized)	weeks	years	% or n (%)	kg/m2	Kg	cm	b.p.m.	% or n (%)	% or n (%)	% or n (%)	years	mm Hg	mm Hg
Barrios V (2007)	Treatment arm	prospective, parallel group, partially open-label/partially randomised，double-blind, double-dummy study	European countries	Olmesartan 20mg	Olmesartan 20mg	HCTZ 12.5mg	325	4 weeks	54.9 ± 10.1	57.8	29.6 ± 5.2	85.7 ± 16.6	–	–	98.5	–	–	6.1 ± 6.4	160.8 ± 14.0	100.2 ± 5.1
	Control arm				Olmesartan 40mg	–	302		54.8 ± 10.9	63.2	29.4 ± 5.2	85.3 ± 16.6	–	–	97.4	–	–	6.2 ± 6.7	160.8 ± 14.2	100.8 ± 5.6
Bönner G (2008)	Treatment arm 01	8-week single-blind run-in phase, followed by an 8-week randomized, double-blind phase with three parallel treatment arms	Germany	Candesartan 16-32mg	Candesartan 32mg	HCT 25mg	659	8 weeks	54.6 ± 10.0	58.7	–	–	–	–	99.2	22.2	5.0	6.3 ± 6.9	153.1 ± 13.6	97.0 ± 5.6
	Treatment arm 02				Candesartan 32mg	HCT 12.5mg	648		54.6 ± 9.9	58.8	–	–	–	–	99.2	22.2	6.2	6.6 ± 6.7	152.6 ± 13.9	96.6 ± 5.3
	Control arm				Candesartan 32mg	–	638		54.9 ± 10.2	56.9	–	–	–	–	98.3	18.8	6.1	7.1 ± 7.4	154.1 ± 14.2	96.8 ± 5.4
Campbell M (2001)	Treatment arm	randomised, parallel-group trial	European countries (Czechoslovakia,	Candesartan 16mg	Candesartan 16mg	HCTZ 12.5mg	164	8 weeks	53.5 ± 9.0	101/164		83.1 ± 15.0		78.4 ± 12.6				5 (0–45)	153.0 ± 13.3	98.2 ± 5.7
	Control arm				Candesartan 16mg	placebo	165/164 (ITT)		52.2 ± 9.2	89/164		83.7 ± 13.5		79.1 ± 11.5				5 (0–38)	153.4 ± 13.1	97.5 ± 5.2

			Hungary, Poland and the UK）																	
Gleim GW (2006)	Treatment arm	multicenter, randomized, double-Blind, parallel-group trial	United States	Lotarran 100 mg	Lotarran 100 mg	HCTZ 12.5mg	147/146 (ITT)	6 weeks	54.5 ± 11.0	85 ± 57.8	31.0 ± 4.9							9.7 ± 8.7	147.5 ± 15.2	97.6 ± 5.9
	Control arm				Lotarran 100 mg	–	145/144 (ITT)		53.1 ± 9.4	83 ± 57.2	32.2 ± 6.2							9.7 ± 8.3	144.4 ± 12.1	97.0 ± 5.3
Hall WD (1998)	Treatment arm	multicentre, doubleblind, randomised, parallel-group trial	United States	Valsartan 80mg	Valsartan 80mg	HCTZ 25mg	177	8 weeks	54.3 ± 10.5	70		91.4 ± 18.0	173.0 ± 10.4					10.1 ± 9.0	152.4 ± 15.2	100.6 ± 5.1
	Treatment arm				Valsartan 80mg	HCTZ 12.5mg	176		53.0 ± 10.7	66		91.3 ± 18.0	172.2 ± 10.4					9.5 ± 8.5	149.6 ± 14.1	99.9 ± 5.1
	Treatment arm				Valsartan 80mg	–	183		52.9 ± 10.4	63		90.3 ± 19.2	170.7 ± 10.2					9.4 ± 8.2	150.1 ± 15.1	100.2 ± 4.9
	Control arm				Valsartan 160mg	–	172		52.5 ± 10.9	63		90.7 ± 18.7	171.7 ± 9.7					9.1 ± 8.1	149.2 ± 15.2	99.8 ± 4.5
Lacourcière Y (2001)	Treatment arm	multicentre, prospective, randomised, double-blind, parallel-group	Canada		telmisartan 80 mg	HCTZ 12.5 mg	246	8 weeks	55.6 ± 10.0	65.0	30.5 ± 5.2			72.9 ± 9.4	95.5			9.0 (0–38)	148.9 ± 14.8	96.4 ± 6.0
	Control arm				telmisartan 80 mg	–	245		55.0 ± 10.7	61.2	31.0 ± 5.4			72.9 ± 8.4	94.3			8.9 (0–47)	148.7 ± 16.1	96.6 ± 5.2
Lacourcière Y (2002)	Treatment arm	prospective, randomized, double-blind, parallel group	Canada		telmisartan 40 mg	HCTZ 12.5 mg	160	8 weeks	53.8 ± 10.1	59.4	30.7 ± 6.2				97.5			7.5 (0–33)	147.1 ± 13.6	95.7 ± 4.7
	Control arm				telmisartan 40 mg	–	167		54.1 ± 9.7	56.9	31.6 ± 6.0				98.8			7.2 (0–39)	146.7 ± 12.7	95.6 ± 4.8
Makita S (2009)	Treatment arm	randomized, parallel-group trial	Japan	candesartan 8 mg/day or valsartan 80 mg/day	telmisartan 40 mg	HCTZ 12.5 mg	32	12 weeks	69.3 ± 9.0	19/32	24.1 ± 2.9	60.4 ± 8.6	158 ± 8	68.7 ± 7.2					162.5 ± 10.9	86.1 ± 8.7
	Control arm				candesartan 8 mg/day or valsartan 80 mg/day	–	32		67.0 ± 7.2	16/32	25.1 ± 2.2	60.1 ± 9.1	154 ± 10	68.4 ± 8.5					160.6 ± 10.9	84.5 ± 7.8

Mallion JM (2003)	Treatment arm	double-blind parallel-group randomized trial	20 countries in Europe, North America and South America		valsartan 160 mg	HCTZ 25 mg	666	8 weeks	55.7 ± 11.2	53.0		82.7 ± 15.2	168.1 ± 9.7	73.4 ± 9.29	89.9				160.4 ± 12.22	101.5 ± 3.99
	Treatment arm				valsartan 160 mg	HCTZ 12.5 mg	670		56.0 ± 11.1	51.5		82.5 ± 17.6	167.6 ± 9.9	73.4 ± 8.97	91.2				160.5 ± 12.68	101.4 ± 4.11
	Control arm				valsartan 160 mg	–	666		55.3 ± 11.2	52.0		82.7 ± 16.7	168.0 ± 9.8	73.0 ± 9.59	90.1				160.2 ± 12.40	101.3 ± 4.06
Phee MY (2015	Treatment arm	multicenter, randomized, active-controlled, double-blind, parallel-group, dose-titration trial	Korea		fimasartan 60/120 mg	HCTZ 12.5 mg	175	4–8 weeks	55.3 ± 9.40	77.98		71.9 ± 9.70	166.8 ± 6.80			54.17		6.20 ± 6.02	150.8 ± 12.70	96.8 ± 5.70
	Control arm				fimasartan 60/120 mg	–	88		53.4 ± 8.20	73.86		71.3 ± 9.10	166.6 ± 7.90			45.45		6.59 ± 5.84	149.4 ± 11.90	96.5 ± 5.40
Ruilope (1996)	Treatment arm	Double-blind, parallel, placebo controlled study	multicenters		Losartan 50 mg	HCTZ 25 mg	80	12 weeks	52.5	42					63			7.6		
	Treatment arm				Losartan 50 mg	HCTZ 12.5 mg	80		51.1	41					54			6.9		
	Control arm				Losartan 50 mg	–	80		52.0	47					58			6.7		
Rump LC (2011)	Treatment arm	two-phase, randomized, double-blind, multi-national, parallel group Phase III study	78 sites in Europe	olmesartan 40mg	olmesartan 40mg	HCTZ 25 mg	140	8 weeks	55.2 ± 8.2	88 (62.9)	30.3 ± 4.7	88.4 ± 15.5				25 (17.9)	6 (4.3)	9.3 ± 8.6	155.4 ± 11.4	98.0 ± 5.6
	Treatment arm				olmesartan 40mg	HCTZ 12.5 mg	278		53.7 ± 9.8	173 (62.2)	29.4 ± 4.3	86.3 ± 15.3				52 (18.7)	25 (9.0)	9.1 ± 9.5	154.0 ± 11.4	97.5 ± 6.0
	Control arm				olmesartan 40mg	–	274		54.1 ± 8.9	159 (58.0)	30.3 ± 4.7	88.3 ± 16.3				53 (19.3)	25 (9.1)	8.9 ± 8.8	155.6 ± 12.4	97.3 ± 5.8
Sachse A (2002)	Treatment arm	multicentre, prospective, randomised, double-blind, parallel group study	European		eprosartan 600 mg	HCTZ 12.5 mg	152	8 weeks	54.9 ± 0.9	75 (49.3)		82.4 ± 1.5	165.7 ± 0.8	73.9 ± 0.7	149 (98.0)				155.3 ± 1.1	99.9 ± 0.4
	Control arm				eprosartan 600 mg	–	157		57.4 ± 0.9	87 (55.4)		81.4 ± 1.3	166.4 ± 0.8	73.0 ± 0.7	152 (96.8)				156.0 ± 1.1	98.9 ± 0.4
Sun NL (2008)	Treatment arm	multicenter, double-blind, double-	China		Valsartan 80mg	HCTZ 12.5 mg	429/419 (ITT)	8 weeks	52.0 ± 10.1	253 (60.4)	25.9 ± 2.9	72.08 ± 11.45	166.4 ± 8.3	73.8 ± 7.2	0	299 (71.4)			143.5 ± 12.4	95.8 ± 4.6

	Control arm	dummy, randomized, active-controlled, parallel-group trial			Valsartan 80mg	–	435/423 (ITT)		52.3 ± 9.9	237 (56.0)	25.8 ± 3.3	71.91 ± 11.86	166.7 ± 8.1	74.1 ± 7.4	0	295 (69.7)			144.0 ± 11.8	95.9 ± 4.7
Toh R (2012)	Treatment arm	prospective, randomized, open-label, comparative multicenter study	Japan		losartan 50	HCTZ 12.5 mg	100/98 (ITT)	12 weeks	69.6±9.6	61.2	24.5±3.5						23.5		153.7±11.1	84.0±11.1
	Control arm				losartan maximal-dose	–	100/95 (ITT)		67.4±11.6	66.3	24.7±3.7						24.2		154.6±11.8	85.4±11.2
Tuomilehto J (2008)	Treatment arm	double-blind, active-controlled, parallelgroup, randomized trial	17 countries across Europe, North America, South America and South Africa		Valsartan 320	HCTZ 25 mg	900	8 weeks	54.4 ± 10.1	57	29.5 ± 4.9			77.7 ± 12.4	88.9				159.9 ± 11.0	100.3 ± 4.0
	Treatment arm				Valsartan 320	HCTZ 12.5 mg	903		53.9 ± 10.0	57	29.5 ± 5.1			77.1 ± 12.0	90.3				160.0 ± 10.9	100.6 ± 3.9
	Control arm				Valsartan 320	–	899		54.2 ± 10.4	57	29.6 ± 4.9			77.1 ± 12.5	89.1				159.4 ± 11.0	100.5 ± 3.9
