# Supplementary figures and images for: Efficacy of ARB/HCTZ Combination Therapy in Uncontrolled Hypertensive Patients Compared with ARB Monotherapy: A Meta-Analysis
Source: Int J Hypertens. 2021 Apr 27;2021:6670183. doi: 10.1155/2021/6670183 (PMC8096582; doi:10.1155/2021/6670183)

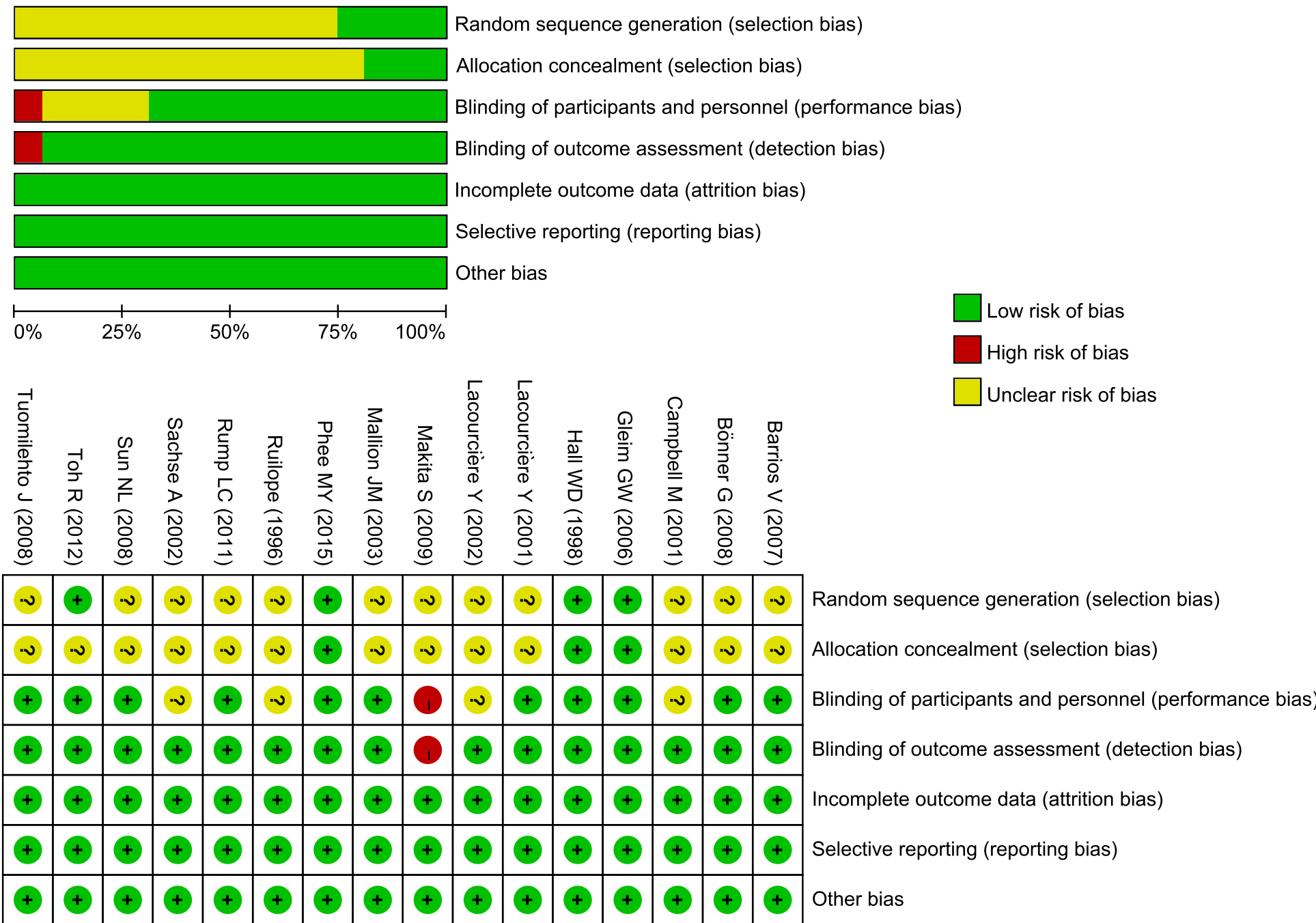

Supplement: Supplementary Materials — Supplementary Table 1: study characteristics of included studies. Supplementary Figure 1: summary of bias of the included studies by the Cochrane risk-of-bias tool. Supplementary Figure 2: funnel plot with Egger's test for assessing the risk of publication bias. Supplementary Figure 3: forest plot for drug-related adverse events (AEs). Supplementary Figure 4: forest plot for severe adverse events (SAEs). Supplementary Figure 5: forest plot for discontinuation due to adverse events (AEs). [file 6670183.f1.zip › 6670183.f1/Supplementary Figure 1 (1).pdf]

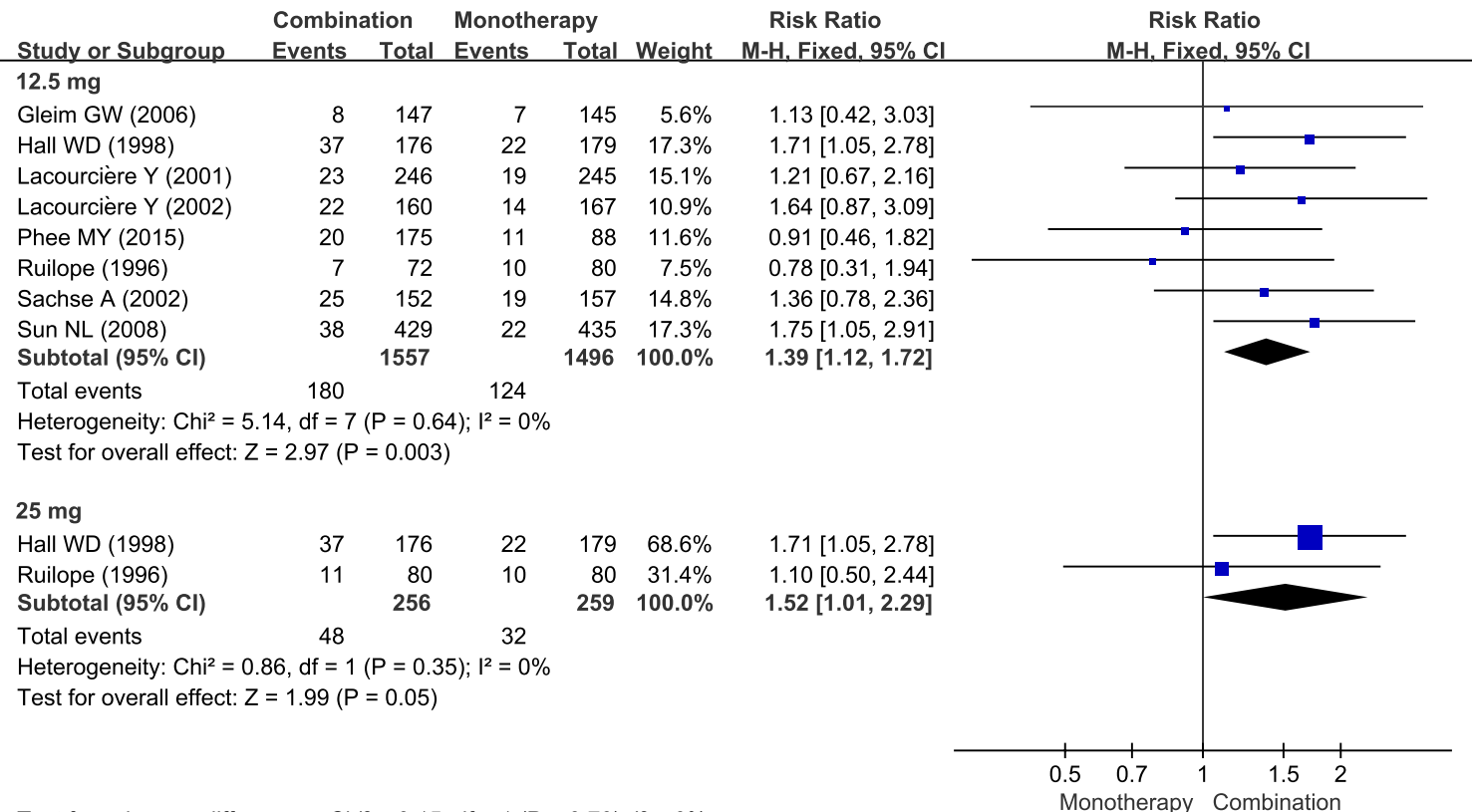

Test for subaroup differences:  $\chi^2 = 0.15$ ,  $df = 1$  ( $P = 0.70$ ).  $I^2 = 0\%$

Supplement: Supplementary Materials — Supplementary Table 1: study characteristics of included studies. Supplementary Figure 1: summary of bias of the included studies by the Cochrane risk-of-bias tool. Supplementary Figure 2: funnel plot with Egger's test for assessing the risk of publication bias. Supplementary Figure 3: forest plot for drug-related adverse events (AEs). Supplementary Figure 4: forest plot for severe adverse events (SAEs). Supplementary Figure 5: forest plot for discontinuation due to adverse events (AEs). [file 6670183.f1.zip › 6670183.f1/Supplementary Figure 3 (1).pdf]

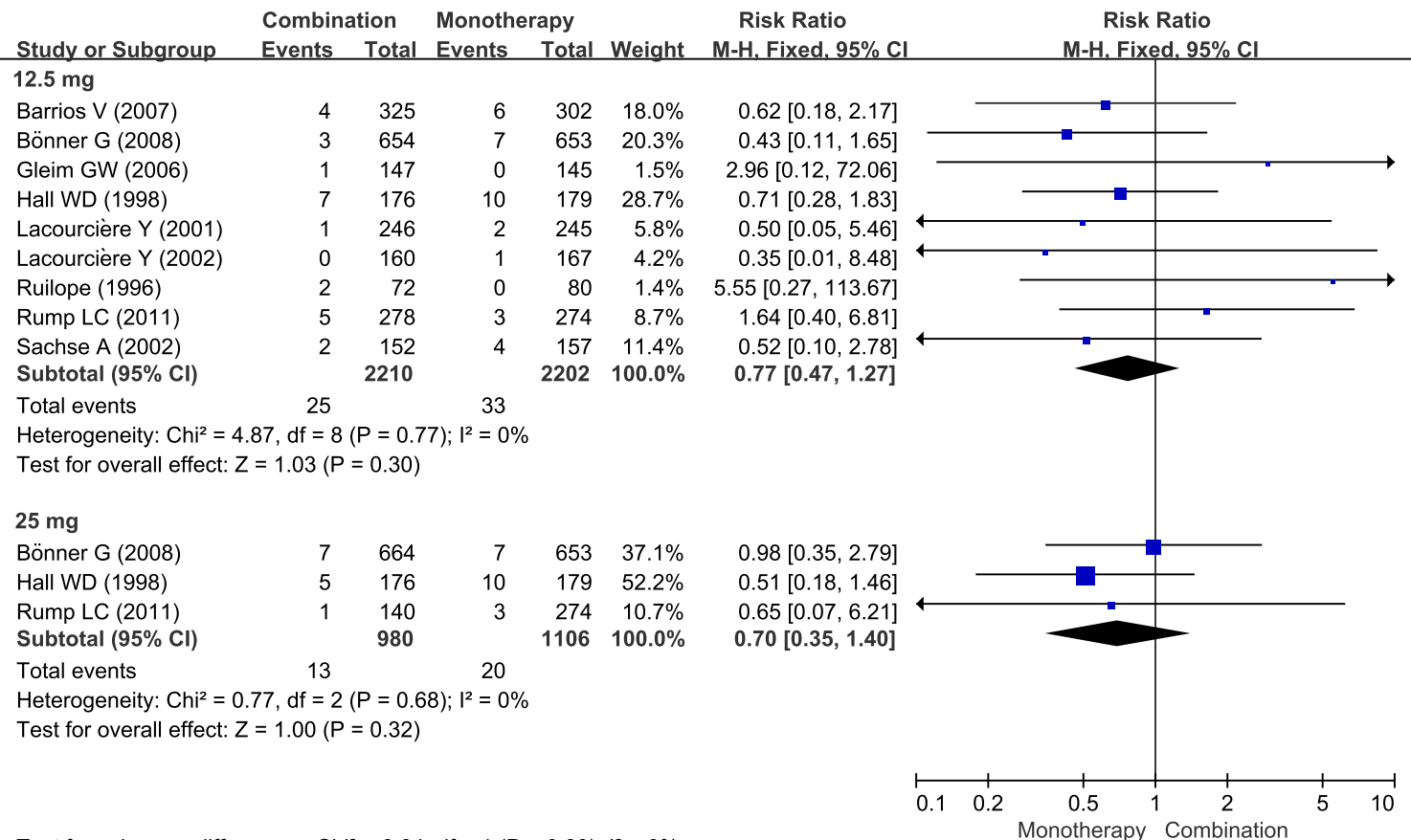

Test for subgroup differences:  $\text{Chi}^2 = 0.04$ ,  $\text{df} = 1$  ( $P = 0.83$ ).  $I^2 = 0\%$

Supplement: Supplementary Materials — Supplementary Table 1: study characteristics of included studies. Supplementary Figure 1: summary of bias of the included studies by the Cochrane risk-of-bias tool. Supplementary Figure 2: funnel plot with Egger's test for assessing the risk of publication bias. Supplementary Figure 3: forest plot for drug-related adverse events (AEs). Supplementary Figure 4: forest plot for severe adverse events (SAEs). Supplementary Figure 5: forest plot for discontinuation due to adverse events (AEs). [file 6670183.f1.zip › 6670183.f1/Supplementary Figure 4 (1).pdf]

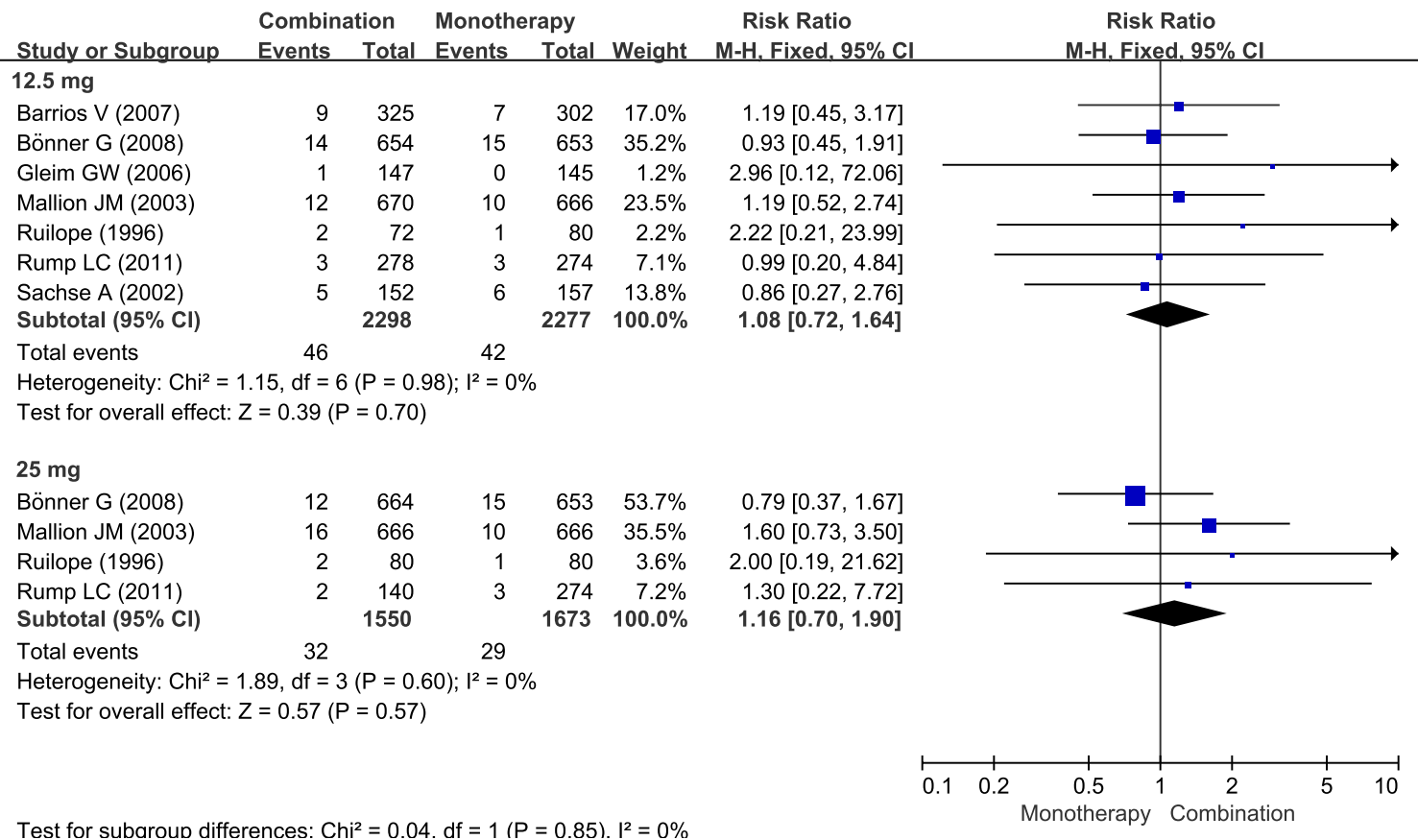

Supplement: Supplementary Materials — Supplementary Table 1: study characteristics of included studies. Supplementary Figure 1: summary of bias of the included studies by the Cochrane risk-of-bias tool. Supplementary Figure 2: funnel plot with Egger's test for assessing the risk of publication bias. Supplementary Figure 3: forest plot for drug-related adverse events (AEs). Supplementary Figure 4: forest plot for severe adverse events (SAEs). Supplementary Figure 5: forest plot for discontinuation due to adverse events (AEs). [file 6670183.f1.zip › 6670183.f1/Supplementary Figure 5 (1).pdf]
